# Assessment and diagnostic accuracy of lymph node status to predict stage III colon cancer using computed tomography

**DOI:** 10.1186/s40644-016-0104-2

**Published:** 2017-01-19

**Authors:** Erik Rollvén, Mirna Abraham-Nordling, Torbjörn Holm, Lennart Blomqvist

**Affiliations:** 1 Department of Molecular Medicine and Surgery, Karolinska Institutet, Department of Radiology, Karolinska University Hospital, Solna, SE - 171 76 Stockholm Sweden; 2 Department of Molecular Medicine and Surgery, Karolinska Institutet, Center for Digestive Diseases, Karolinska University Hospital, Stockholm, Sweden

**Keywords:** Colon cancer, Computed tomography, Staging, Stage III, Lymph nodes

## Abstract

**Background:**

To study different imaging criteria for prediction of lymph node metastases (Stage III disease) in colon cancer using CT.

**Methods:**

In a retrospective setting, 483 consecutive patients with histology proven colon cancer underwent elective primary resection during 2008–2011, a cohort of 119 patients were included. Contrast enhanced CT examinations, in portal-venous phase, were reviewed with assessment of the number of lymph nodes, their anatomical distribution, size, size ratio, internal heterogeneity, presence of irregular outer border and attenuation values. Sensitivity, specificity, PPV and NPV for each studied criteria for prediction of stage III disease was calculated.

**Results:**

According to histopathology 80 patients were stage I-II and 39 were stage III. Of the studied CT-criteria for lymph node metastases per patient, internal heterogeneity in at least one lymph node resulted in the best performance with sensitivity, specificity, PPV and NPV of 79, 84, 70 and 89%, Odds ratio (OR) 20. Presence of irregular outer border resulted in a sensitivity, specificity, PPV and NPV of 59, 81, 61 and 82%, OR 6.2. If both internal heterogeneity and/or irregular outer border was used as a criterion this resulted in a sensitivity, specificity, PPV and NPV of 85, 75, 62 and 91%, OR 16.5. None of the size criteria used were predictive for stage III disease.

**Conclusions:**

When performing preoperative CT in patients with colon cancer, the imaging criteria that allow best prediction of stage III disease on CT are either presence of at least one lymph node with internal heterogeneity or internal heterogeneity and/or irregular outer border. These criteria have to be validated in a prospective study.

## Background

Colon cancer is the third most common malignancy in the western world. In Sweden the incidence is increasing with an aging population while the mortality is slowly decreasing [1].

The treatment is surgical removal of the tumour containing segment of the bowel together with local and regional lymph nodes. In addition, adjuvant chemotherapy is standard treatment for patients with stage III disease and in some patients with stage II disease, depending on presence of additional histological risk factors.

Well-known important prognostic factors in colon cancer are tumour stage (T), extramural vascular invasion (EMVI) and lymph node involvement (N) [2]. Even the total number of harvested lymph nodes at surgery and lymph node ratio (the ratio between lymph node metastases and examined lymph nodes, LNR) assessed by the pathologist has a prognostic importance [3–5].

A complete preoperative evaluation of patients with colon cancer includes staging of the primary tumour and evaluation of distant metastases in the liver and lungs with computed tomography (CT). In recent years, some studies advocate and support the use of CT also for local staging of colon cancer including treatment planning and selection of patients for neoadjuvant treatment [6–8]. If selection of patients for neoadjuvant treatment is being used routinely in the clinic, pretreatment knowledge of regional lymph node involvement will be even more important.

To date, there are no validated imaging criteria for the assessment of lymph node metastases in colon cancer. Previous studies have applied different criteria based on either size and/or morphology. Lymph node size >1 cm, short-long axis diameter ratio, internal heterogeneity (IH), irregular outer border (IOB), attenuation values >100 Hounsfield units (HU) and cluster of three or more normal sized lymph nodes, or any combination of the above, have all been used as a single criterion or combined criteria [6, 9–14]. In a systematic review by Leufkens et al. including 753 patients with colon cancer in altogether 11 studies a sample sized weighted sensitivity and specificity of CT for N-staging of 76 and 55% was reported [15]. Most of the studies included were performed with older CT-technology that is no longer used and that does not allow true multiplanar assessment. Furthermore, the studies did not consider the distribution and location of lymph nodes within the colonic mesentery adjacent to the cancer as a potential marker of lymph node involvement.

Today, when CT scanners are configured with multiple rows of detectors, multiplanar assessment can be performed allowing for more detailed assessment of size and morphology of pathological lesions [16].

The aim of this study was to assess whether the number of lymph nodes, their anatomical distribution, size, size ratio, internal heterogeneity, irregular outer border and attenuation values on preoperative CT, either alone or in combination, were predictive for stage III disease.

## Methods

From the Swedish colorectal cancer registry (SCRCR), 483 consecutive patients having a histology proven colon cancer and operated between the years 2008 and 2011 and examined with abdominal CT (64 detector CT-scanner) before surgery at our institution were included. A cohort of 119 patients was determined, after the following patients were excluded: insufficient CT examination (no iv contrast, CT-Colonography, no 64-slice) including examination performed outside the University hospital (*n* = 80), patients having emergency colonic surgery (*n* = 78), patients with T4 tumours (*n* = 68), metastatic disease (*n* = 67), no detectable tumour on the preoperative CT (*n* = 18), co-malignant disease (*n* = 15), CT examination >60 days prior to surgery (*n* = 13), patients with neo-adjuvant chemotherapy (*n* = 11), previous colon cancer surgery (*n* = 9), CT after treatment with colon stenting (*n* = 3) and perforated tumour or abscess (*n* = 2).

In the remaining cohort with histologically proven colon cancer there were 63 women and 56 men with a median age of 69 (range 32–91 years).

Most of the tumours were located in the sigmoid colon (Table 1). The majority of the tumors were classified on histopathology as T3 tumours (Table 1). Thirty-nine out of 119 patients had lymph node positive (stage III) disease (28 patients N1 and 11 patients N2) (Table 1). The median age for patients with stage I-II disease was 70 years, and the median age for patients with stage III disease was 63 years.

**Table 1:** Demographics table of 119 patients/tumours

Characteristics	Number (%)
Sex (female/male)	63/56
Age (median)	69 (32–91)
Histopathological evaluation	
Tumour localization	
Caecum	23 (19%)
Ascending colon	22 (18%)
Hepatic flexure	8 (7%)
Transverse colon	11 (9%)
Splenic flexure	4 (3%)
Descending colon	6 (5%)
Sigmoid colon	45 (38%)
Tumor Stage	
T1	10 (8%)
T2	16 (13%)
T3	93 (78%)
Positive lymph node status	
T1 tumours	2/10 (20%)
T2 tumours	2/16 (12%)
T3 tumours	35/93 (38%)
Stage	
Stage I	22 (18%)
Stage II	58 (49%)
Stage III	39 (33%)
Lymph nodes, total number	
Harvested lymph nodes PAD	2542
Positive lymph nodes PAD	123
CT evaluation	
Detected lymph nodes ≥4 mm/tot	442/1312 (34%)
Region 1	261/835 (31%)
Region 2	161/389 (41%)
Region 3	20/88 (23%)

All patients had preoperative investigations with CT of the abdomen with intravenous contrast (0.5 mg Iodine/kg) in portal-venous phase (delay 90 s) on one of four different 64 slice CT scanners (Lightspeed VCT, General Electric, Milwaukee, USA). All examinations were performed at 120 kV and with tube current modulation. For abdomen the median dose length product (DLP) was 583 mGy-cm (range 393 to 878 mGy-cm). Median pitch factor was 1.375 (range 0.516 to 1.50). Medium noise index was 30 (range 26 to 42). The variation in both pitch factor and noise index are due to differences in the four CT scanners that were used. In 56 CT examinations arterial phase imaging of the abdomen at the tumour location and liver was also performed. After the examination, reformatted images in axial, coronal and sagittal planes with 5 mm thickness (increment 2.5 mm) were routinely generated together with the original (thin slices) 0.625 mm images.

### CT evaluation

All CT examinations were retrospectively reviewed by one radiologist (E.R.) with more than 15 years of experience in cross sectional imaging of colorectal cancer and blinded for the histology and surgical reports. Examinations were assessed according to a dedicated evaluation proforma.

All measurements and assessments were performed on a Sectra Workstation IDS7 (version 15.1.14.41) using the 5 mm reformatted images with 2.5 mm increment. The original thin slices (0.625 mm) were used for detection of small lymph nodes (≤4 mm).

#### Anatomical distribution

**Fig. 1:**
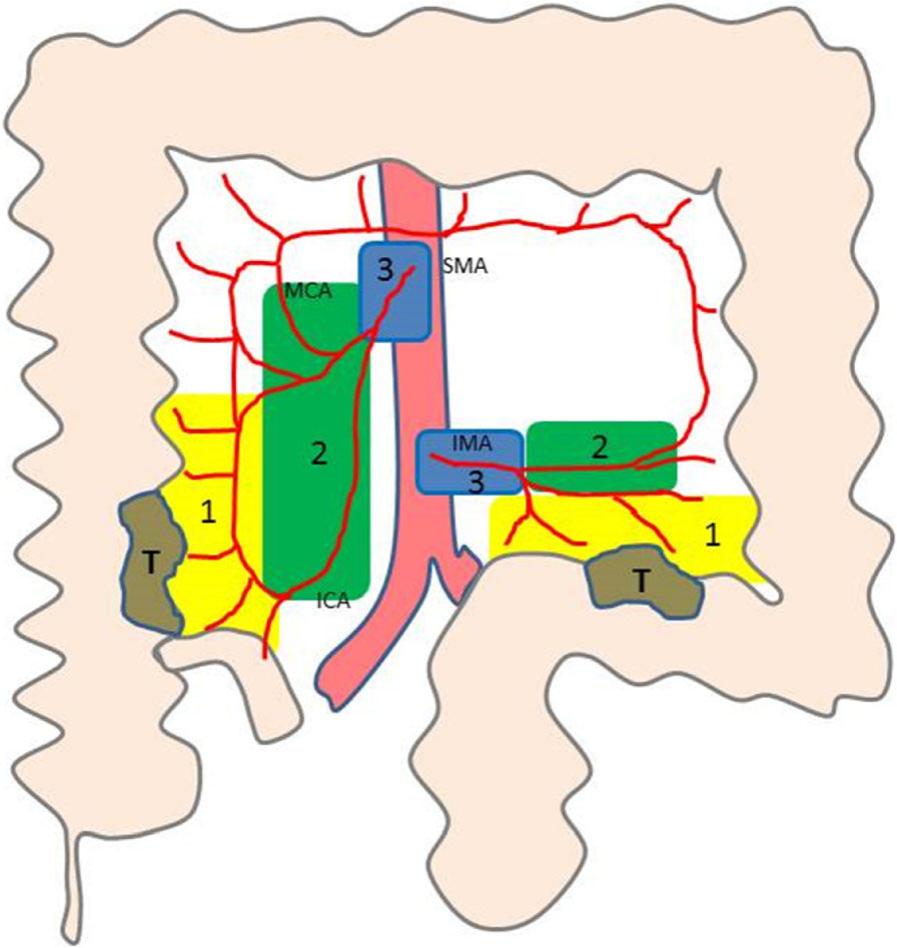
Assessment of right and left sided tumours (T) and their corresponding anatomical distribution of lymph nodes. Region 1 (*yellow*) is +/− 5 cm oral and aboral near the tumour site and approximately 3 cm along the feeding arterial branch to the nearest arterial vessel division. Region 3 (*blue*) is the undivided artery from the aorta (superior mesenteric artery (SMA) or inferior mesenteric artery (IMA)) to the first artery division. Region 2 (*green*) is between regions 1 and 3

The colonic mesentery, 5 cm oral and aboral from the tumour site, was divided in three anatomical regions (region 1–3), as a modified variant of the guidelines of the Japanese Society for Cancer in the Colon and Rectum [17]. Region 1 was defined as the region most adjacent to the tumour (+/−5 cm) and 3 cm proximal along the vessels to the branch artery divides covering the pericolonic and marginal lymph nodes. Region 3 was defined as the most proximal part of the mesentery including the undivided mesenteric artery from the aorta (proximal lymph nodes). Region 2 was defined as the region between region 1 and 3 (Fig. 1).

#### Number, size and size-ratio of lymph nodes

All lymph nodes ≥2 mm in size were separately registered in total and in each anatomical region. For lymph nodes ≥4 mm in shortest diameter, the short axis and the long axis were also separately measured and the ratio between the short and long axis diameter was calculated. The size ratio (ratio between two orthogonal (short/long) axis diameters) was used to test whether a more rounded shape was predictive for metastasis. A >0.8 ratio between diameter was used as cut off point according to a previous study [18]. The presence of a cluster (within a range of the lymph node diameter) of three or more lymph nodes was also separately noted in every region.

#### Internal heterogeneity and irregular outer borders

**Fig. 2:**
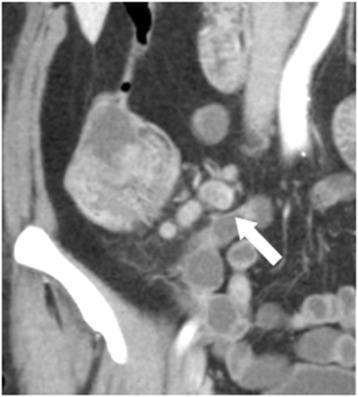
Coronal reformation (5 mm section post iv contrast portal phase) CT image illustrating the apperance of 10 × 15 mm lymph node (*white arrow*) in region 1 with internal heterogeneity thus well defined borders in a patient with pT3 tumour in the ascending colon. At histopathology, 4 metastatic lymph nodes out of 43 were harvested

**Fig. 3:**
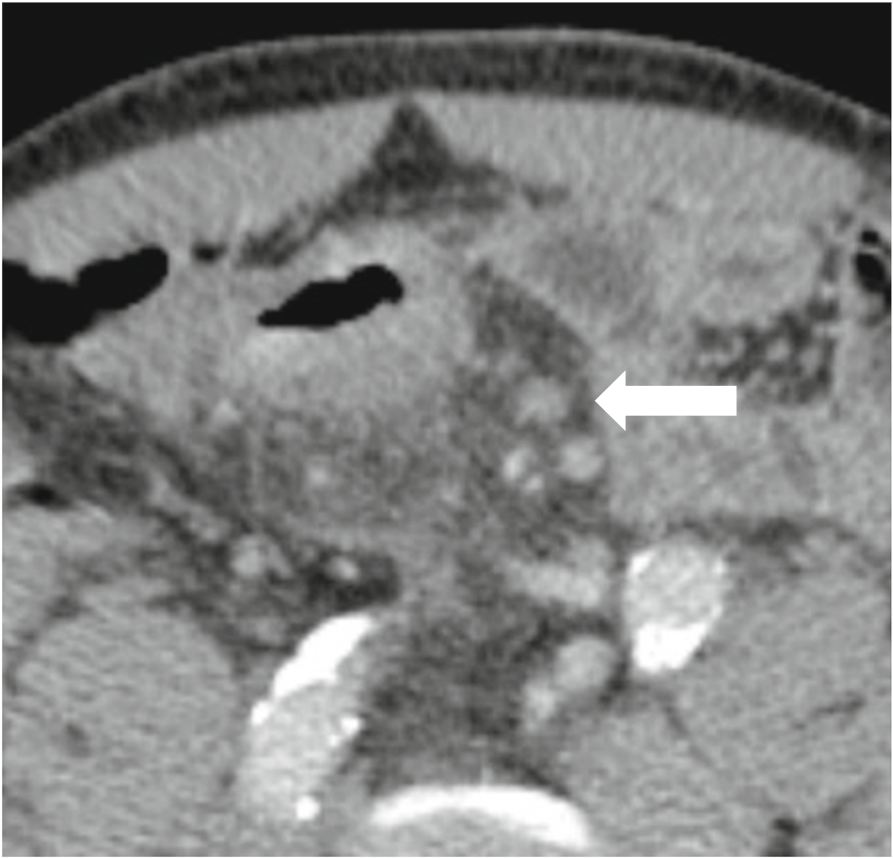
Transaxial reformation (5 mm section post iv contrast portal phase) CT image illustrating the apperance of a 7 × 7 mm mesocolic lymph node (*white arrow*) in region 2 with irregular outer border and internal heterogeneity in a patient with a pT3 tumour in the sigmoid colon with 1 metastatic lymph nodes out of 16 harvested at histopathology

As possible morphological predictors of metastases, the internal heterogeneity (IH, mixed attenuation within the lymph node) as well as the irregular outer border (IOB, indistinct demarcation of the lymph node) were evaluated both on reformatted and thin sections (Figs. 2 and 3).

#### Attenuation values

Attenuation measurements of each lymph node in the portal venous phase and, when available, in the arterial phase were also performed. All density measurements (using HU) were performed by placing as large a region of interest (ROI) as possible (>2 mm^2^) on the lymph node in the portal venous phase and in the arterial phase when available. Attenuation values of ≥50 and ≥100 HU in the portal venous phase were separately noted as well as ratio portal venous/arterial phase. Inhomogeneous contrast enhancement as indicative for tumour involvement was separated from either presence of a fatty lymph node hilum or a contrast filled vessel in the vicinity of a lymph node.

#### Surgery

All patients in the study were operated in an elective setting and according to colorectal surgery praxis. The resection of colon cancer was made by clear lateral margins, resection of the loco-regional lymph node bearing mesentery.

#### Histopathology

Histopathology was performed according to standard procedures at the university hospital pathology department by a specialized GI pathologist (initially using TNM version 6 and later TNM version 7) [19, 20]. From the pathologists’ original report the T- and N-stage, the total number of harvested and metastatic lymph nodes served as reference standard.

#### Statistics

Data were evaluated using statistical analysis software SPSS, IBM. Descriptive statistics were applied to the different lymph node characteristics calculating sensitivity, specificity, PPV, NPV and Odds ratio for the prediction of stage III disease. Mann–Whitney U test were used to test significance which was set to *p* ≤ 0.05. Univariate and multiple logistic regression analyses were performed for categorical data. Receiver operating characteristics (ROC) and area under the curve (AUC) were used to compare the optimal lymph node size criterion.

## Results

### Patients and histopathology

The median time interval between pre-operative CT examination and surgery was 28 days (range 4 – 59 days), mean 29 days (standard deviation 13 days).

A total of 2542 lymph nodes were harvested (median 19 lymph nodes/patient, range 4–69) and of those 123 were assessed as metastases (median, 2 lymph nodes/patient, range 1–10). The change from TNM 6 to TNM 7 did not affect the result.

#### CT evaluation

### Number, anatomical distribution, size and size-ratio of lymph nodes

At CT, most of the lymph nodes were located in region 1. Region 2 had higher proportion of lymph nodes ≥4 mm (41%) compared to the other regions (Table 1). The mean number of lymph nodes found was 7.2 for pT1 tumours, 8.6 for pT2 tumours and 11.8 for pT3 tumours (not shown in Table).

### Evaluation of lymph nodes

**Fig. 4:**
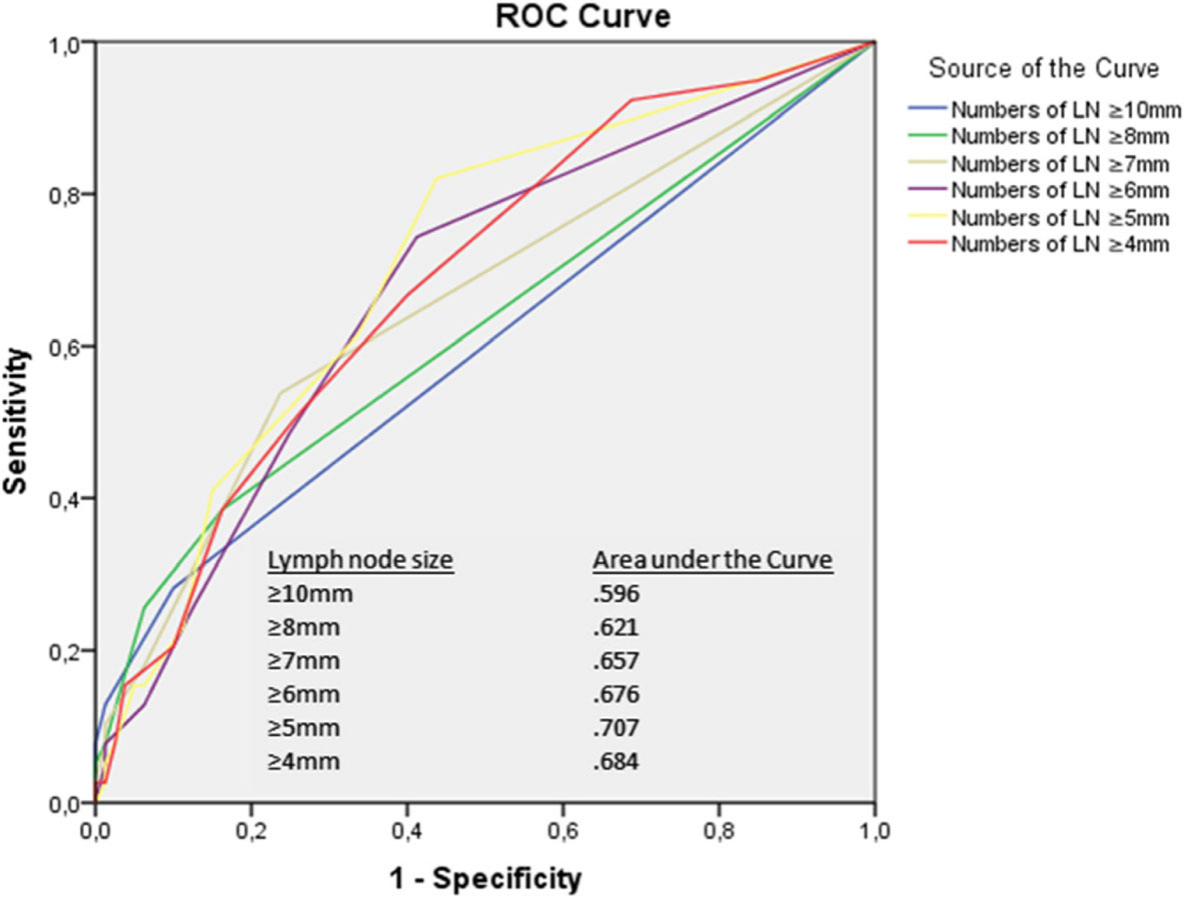
Size criteria (≥4, 5, 6, 7, 8, 10 mm) in shortest diameter according to CT presented as receiver operating characteristics, ROC, and Area under the *curve*, AUC

Using size thresholds of ≥4, 5, 6, 7, 8 and 10 mm as criteria for lymph node metastases, the results are presented as a ROC-curve in Fig. 4.

Size ratio with a cutoff point of 0.8 had an overall sensitivity and specificity of 85 and 30% (Table 2). If divided into the three anatomical regions, the sensitivity and specificity were as follows: region 1, 80 and 35%; region 2, 54 and 74% and region 3, 5 and 94%, respectively.

**Table 2:** Sensitivity, specificity, PPV and NPV (%) for the different CT characteristics of lymph nodes > 4 mm in shortest diameter

Variables	Number	Sensitivity	Specificity	PPV	NPV	OR	*p*-value
Size ≥5 mm	287	90	31	39	86	1.33	0.002
Size ≥10 mm	29	28	90	58	72	2.67	0.009
Ratio cut off 0.8	244	85	30	37	80	2.36	0.090
Internal heterogeneity (IH)	94	79	84	70	89	20.0	<0.001
Irregular outer border (IOB)	73	59	81	61	82	6.23	<0.001
IH and/or IOB	67	85	75	62	91	16.5	<0.001
HU ≥50	396	95	20	37	89	4.63	0.049
HU ≥100	81	44	68	40	71	1.60	0.239
Cluster of three	14	13	89	36	68	1.16	0.803

*Note*: *HU* Hounsfield units, *OR* Odds ratio

### Internal heterogeneity and irregular outer border

Forty-four out of 119 patients had at least one (range 1–6) lymph node with internal heterogeneity according to CT and a total number of 94 lymph nodes with this morphological feature were detected. Compared to histopathology, the sensitivity and specificity for predicting stage III disease with this criterion was 79 and 84%, respectively, *p* ≤ < .001, OR = 20 (Table 2). If divided by anatomical region, the sensitivity and specificity were as follows: region 1, 64 and 91%; region 2, 51 and 92% and region 3, 3 and 97%, respectively.

Thirty-eight patients had at least one (range 1–8) lymph node with an irregular outer border.

Compared to histopathology, lymph nodes with an irregular outer border showed sensitivity and specificity for prediction of stage III disease of 59 and 81%, respectively, *p* ≤ < .001, OR = 6.3 (Table 2). If divided by anatomical region, the sensitivity and specificity were as follows: region 1, 49 and 89%; region 2, 33 and 91%, and region 3, 0 and 99%, respectively.

In patients with at least one lymph node with internal heterogeneity *and* a lymph node with an irregular outer border, regardless of location, the sensitivity and specificity for stage III disease was 54 and 90%, respectively.

Patients with any lymph node showing internal heterogeneity and/or irregular outer borders, meaning that either one of the criteria were present or both in combination, showed an overall sensitivity and specificity for stage III disease of 85 and 75%,respectively, *p* ≤ < .001, OR = 16.5 (Table 2).

### Contrast enhancement

The overall sensitivity and specificity prediction of stage III disease for lymph nodes having a HU value ≥50 or ≥100 post contrast in portal venous phase were, 95 and 20%, and 44 and 68%, respectively (Table 2).

### Cluster of three or more normal shaped and sized lymph nodes

This criterion resulted in an overall sensitivity of 13% and a specificity of 89% (Table 2).

### Combination of different variables using multivariate logistic regression analyses

**Fig. 5:**
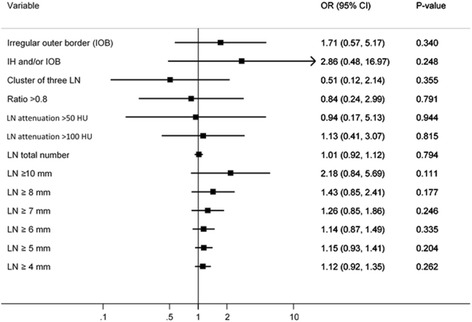
Multivariate logistic regression analyses for odds ratio of different lymph node characteristics when internal heterogeneity is included in the model

The strongest predictor for stage III disease in our study was internal heterogeneity. No other variable contributed significantly when the variable internal heterogeneity was included in the multivariate regression model (Fig. 5).

## Discussion

To our knowledge, this report is the first to study previously reported criteria for lymph node metastases on CT separately or in comparison as a predictor for stage III disease in colon.

Of all the studied imaging criteria in this study, morphological criteria was superior to size criteria. Internal heterogeneity and irregular outer borders were the two variables that display best, both alone or combined, with reasonable sensitivity and specificity. The combination of internal heterogeneity and/or irregular outer borders still resulted in a moderate sensitivity of 85% and specificity of 75%. The strongest predictor in our study for stage III disease was internal heterogeneity both alone or combined with other variables (Fig. 5). Our results using CT are inferior, but for sensitivity comparable with previous work by Brown et al., which used morphological predictors for mesorectal lymph node status in magnetic resonance imaging (MRI) of rectal cancer where mixed signal intensity or irregular border resulted in a sensitivity of 85% and specificity of 97% [21].

The majority (64%) of lymph nodes that were detected, regardless of N-stage, were located in region 1, but presence of lymph nodes in this region were not predictive for nodal disease (stage III) (*p* = 0.182). In region 2 there was a slightly higher proportion of lymph nodes in favour of stage III (160 out of 471 lymph nodes (34%)) vs stage II (229 out of 841 lymph nodes (27%)) (*p* = 0.006). In the whole cohort, only 39 patients had lymph nodes in region 3, and there was no difference between the two groups regardless of T-stage. The high specificity in region 3 for the criteria internal homogeneity, irregular outer border and size ratio cut off <0.8 is due to a very limited number of lymph nodes fulfilling those criteria.

In a study, with 106 patients and up to date computed tomography technique using >1 cm and/or cluster of ≥3 lymph nodes as criteria for nodal disease resulted in a sensitivity of 71% and specificity of 41% [22]. In the present study, only 19 patients had lymph nodes ≥10 mm and only 14 patients had lymph nodes in a cluster of three, thus reducing the impact of these criteria for prediction of stage III.

Regarding lymph node size, lymph nodes >5 mm and/or irregular outer border were considered positive for nodal disease in the study by Dighe et al. with sensitivity and specificity of 64 and 53%, respectively [8]. In our study, this criterion showed a similar sensitivity of 56% but a higher specificity of 84%. We have no explanation for this difference.

Size criteria alone can really be questioned and not supported by our study. It has been reported that up to 70% of lymph nodes with metastases in colorectal cancer are ≤5 mm in diameter [23]. The majority of the detected lymph nodes (*n* = 870, 66%) in our study were <4 mm and thus could not be further assessed because of their small size and uncertainty in characterization using CT.

In the study of Kwak et al., a combination of criteria regarding assessment of lymph node metastases, including a cluster of more than three nodes along the loco-regional vascular pedicle, spiculated and indistinct node borders, and a mottled heterogeneous pattern were all integrated in the assessment and together with the size threshold of ≥10 mm reporting a sensitivity and specificity of 87 and 29%, respectively, with CT alone [24]. The specificity was surprisingly low maybe due to the large threshold size. The results with positron emission tomography/computed tomography (PET/CT), with a slightly different setting of defined criteria, did not markedly improve the overall results with a sensitivity and specificity of 66 and 60%, respectively.

Other studies using fluoro-2-deoxy-D-glucose-PET (FDG-PET) reported low sensitivities (29–37%) but higher specificity (87%) for nodal staging, suggesting that PET/CT is of limited additional value in detecting metastatic regional lymph nodes surrounding the primary tumour due to high false negative rate [25, 26]. Some authors argues that routine use of PET/CT can alter or change the management in stage III patients (6.5%) and stage IV patients (12.7%) while other authors claim that it does not [27, 28].

Regarding contrast enhancement/attenuation features, this does not seem to increase diagnostic accuracy. Arterial or portal venous phase attenuation post contrast was not predictive for stage III disease in this study. The differences in attenuation between arterial and portal-venous phase was not predictive for stage III disease.

Furthermore, regarding lymph node size ratio, Kanamoto et al. reported both high sensitivity and specificity of 87 and 80%, respectively, using the criteria of cutoff point of 0.8 or greater in short/long axis diameter ratio measured in the axial plane of the CT images [18]. In this current study the reformatted images were used to measure the true short and long axis and found similar sensitivity (85%) for this criterion but much lower specificity (30%) due to a high rate of false positive findings (70%). Benign lymph nodes can be either oval or rounded, which is a limitation using this criterion.

The use of CT assessed lymph node status alone as predictor of prognosis is still premature. If selection for neoadjuvant chemotherapy was made by the best combination of criteria (heterogeneity and/or irregular border) in this study, 6 patients out of 39 (15%) would potentially be undertreated and 20 patients out of 80 (25%) overtreated. This emphasizes the need to decide on such treatment based on other prognostic factors or use them in combination with the assessed lymph node status.

The strength of this study, beside the use of several imaging criteria for lymph node metastases, is the homogenous patient cohort; all examined with 64-multidetector CT and all having primary surgery allowing histopathology of the resected specimen as reference.

Limitations of the study were the retrospective setting. The assessment by only one observer could reduce the robustness and reproducibility of the imaging criteria. These criteria must be validated in a multi reader setting. There were also a limited number of patients with some of the criteria. Furthermore, the histopathological tissues were not reevaluated and there was no possibility to match individual lymph nodes between imaging and histopathology. Another possible limitation was that we also excluded T4 tumours in the study. We argue that the high rate of lymph node metastases in this group of patients (around 37%) is likely to bias the radiologist when looking on CT and assessing the lymph nodes. No texture analysis software was available at the time of the study. We believe that texture analysis may have a role in the context of characterizing regional lymph nodes on CT in colon cancer although the approach in this setting is rather unexplored and may be subjected to a separate study.

## Conclusion

Various imaging criteria for lymph node metastases on CT have been used in previous literature. The results of the present study do not support use of any the commonly used criteria for lymph node metastases. When performing preoperative CT in patients with colon cancer, the imaging criteria that allow the best prediction of stage III disease are either the presence of at least one lymph node with internal heterogeneity or the presence of at least one lymph node with internal heterogeneity and/or irregular outer border. These criteria have to be validated in prospective studies.
